# Avoiding waste of research resource: cohort study of publication rate for funded studies from a major UK research funder

**DOI:** 10.1186/1745-6215-14-S1-P95

**Published:** 2013-11-29

**Authors:** Sheila Turner, David Wright, Rebecca Maeso, Andrew Cook, Ruairidh Milne

**Affiliations:** 1 National Institute for Health Research, Evaluation, Trials and Studies Coordinating Centre (NETSCC), University of Southampton, Southampton, UK; 2 Wessex Institute, University of Southampton, Southampton, UK

## Objectives

Failure to publish findings from research is a significant area of research waste. It has previously been suggested that potentially over 50% of studies funded are never published. This study aimed to investigate what percentage of NIHR HTA programme funded projects have published their final reports in the programme's journal Health Technology Assessment, (the monograph series); and to explore reasons for non-publication.

## Methods

Study included all NIHR HTA projects with a planned submission date for their draft final report (DFR) for publication in the journal series, on or before 9th December 2011. Projects were classified according to whether they had published or not. Reasons for non-publication were investigated.

## Results

628 projects were included: 582 (92.7%) had published a monograph; 19 (3.0%) were expected to publish a monograph; 13 (2.1%) were discontinued studies and would not publish; 12 (1.9%) submitted a report which did not lead to publication as a monograph; and two (0.3%) did not submit a report. Reasons why projects failed to complete included: failure to recruit; issues concerning the organisation hosting the research; drug licensing issues; staffing issues; and access to data.

Overall 95.7% of HTA studies either have published or will publish a monograph: 94% for those commissioned in 2002 or before and 98% for those commissioned after 2002.

## Conclusions

Monographs are published for a very high percentage of NIHR HTA projects. Advantages of this model of publishing include: avoidance of publication bias and research waste; while enhancing accessibility and transparency of findings.

